# Challenging rigidity in Anorexia (treatment, training and supervision): questioning manual adherence in the face of complexity

**DOI:** 10.1186/s40337-021-00460-2

**Published:** 2021-08-21

**Authors:** Annaleise Robertson, Chris Thornton

**Affiliations:** 1 The Children’s Hospital at Westmead, Sydney, Australia; 2 The Redleaf Practice, Sydney, Australia

**Keywords:** Eating disorders, Anorexia nervosa, Adherence, Manuals, Training, Supervision, Evidence-based practice, Therapeutic alliance

## Abstract

**Background:**

Anorexia Nervosa is a debilitating illness. While there have been many advancements to treatment protocols and outcomes for people with eating disorders, the field acknowledges there remains considerable room for improvement. This timely Special Edition of the Journal of Eating Disorders has invited those of us in the field to consider a range of topics in aid of this task, including potential modifications and implementation of evidence-based practice, specific and common psychotherapy factors, treatment manuals, adherence and individualising treatment approaches for individuals and families.

**Body:**

In this paper, we briefly outline the key manualised treatments currently available to treat children, adolescents and adults with Anorexia Nervosa, considering the benefits, potential reasons for adaptations and limitations. We then review the current evidence for training strict adherence to treatment manuals which is often a key focus in training and supervision, questioning the association of increased treatment adherence with improved therapeutic outcome. We then summarise some key evidence behind other therapeutic factors which have been demonstrated to affect outcome regardless of which manual is implemented, such as readiness to change and therapeutic alliance.

**Conclusion:**

The paper concludes with implications and considerations for future research, clinical guidelines, training and supervision, highlighting the need to consider the therapeutic relationship and processes alongside manual content to conduct best evidence-informed practice.

## Plain English summary

While there have been many advancements to treatment options and recovery rates for people with eating disorders, the field acknowledges there remains a long way to go. The development of treatment manuals for clinicians to use has many benefits, including promoting rigorous research, clear training and clinical guidelines, broader dissemination and accessibility, a common language for professionals, platforms from which to research necessary adaptations, and a set of core treatment principles. While it is often assumed that strict adherence to manuals will lead to the best treatment outcomes, research tells us a different story; that working flexibly and collaboratively with service users in an individually tailored way, focusing on meeting them where they’re at, and building trust and understanding between them may actually be the best way to improve treatment experience and outcomes. This paper highlights the need for this way of working to be embraced as a crucial part of evidence-informed practice, with some suggestions for further research, treatment guidelines, training and supervision provided.

## Background

Anorexia Nervosa (AN) is a debilitating illness. While there have been many advancements to treatment protocols and outcomes for people with eating disorders, the field acknowledges there remains considerable room for improvement [1]. Positively, manualised treatments such as Family Based Treatment (FBT) [2, 3] and Enhanced Cognitive Behaviour Therapy (CBT-E) [4, 5] have enabled a wider breadth of outcome research and clinical training to be implemented, and the development of fundamental principles for clinical practice and training standards both nationally [6–8] and worldwide [9, 10]. In this paper, we explore the evidence for training strict adherence to treatment manuals, considering the benefits, potential reasons for adaptations and limitations. We then outline evidence behind other therapeutic factors which have been demonstrated to impact outcome regardless of which manual is implemented, such as readiness to change and therapeutic alliance. We conclude with implications and considerations for current research, clinical guidelines, training and supervision.

### Manualised treatments

Key manualised treatments have been developed for the psychological treatment of eating disorders. For children and adolescents, the National Institute for Health and Care Excellence (NICE) guidelines [9] recommend family therapy, of which manualised versions include FBT [3] and Family Therapy for Anorexia Nervosa (FT-AN) [11]. If family therapy is not appropriate, individual options include CBT-E adapted for adolescents [12] or Adolescent-Focused Therapy (AFT) [13].

For adults with AN, the NICE guidelines suggest Cognitive Behaviour Therapy (CBT) [4, 5], Maudsley Anorexia Nervosa Treatment for Adults (MANTRA) [14] or Specialist Supportive Clinical Management (SSCM) [15], all of which have accompanying manuals. While not listed as a first line option, Focal Psychodynamic Therapy [16, 17] is also suggested as an appropriate alternative if the above options are deemed unsuitable or ineffective. There are also other versions of psychotherapy, such as Interpersonal Psychotherapy (IPT), which achieve equivalent outcomes [18]. While there is certainly a sound evidence base from which to continue to research treatment, training and supervision, the quality of this evidence base remains a debated topic [19, 20].

There are clear benefits to the use of treatment manuals within the eating disorders field and within psychology more generally. One key benefit is they promote the ability to engage in more rigorous research, such as randomised controlled trials (RCTs) which, alongside meta-analyses, are considered the highest forms of evidence [21]. Furthermore, manuals provide clear guidelines for training therapists or those inexperienced within the field, and their accessibility enables broader dissemination beyond in-person or on the job training. Similarly to other manuals like the Diagnostic and Statistical Manual of Mental Disorders (DSM) [22], treatment manuals also help professionals to adopt shared language. This is important to aid communication and a consistent approach within and between treatment teams, which is particularly important given the need for multidisciplinary care for those with AN [23].

### Adherence and drift in treatment manuals

Therapist adherence is defined as the accuracy with which the specified protocols of a manualised treatment are implemented in the clinical setting [24]. It is argued to be a key component in training and providing evidence-based treatment, and is a large focus in research, training and supervision [25]. However, it is also recognised that therapists commonly deviate substantially from empirically supported protocols in psychotherapy both generally, and within the eating disorders field specifically [26]. Such deviations from the manuals are labelled as non-adherence or therapist drift, which is defined as where therapists either actively decide to ignore or passively avoid key elements of treatment manuals [27–31]. Non-adherence and therapist drift often have negative connotations [28–30]. Non-adherence is cited as a reason for ‘failed Family Based Treatment’ [29]. An implication of this ‘failure’ is that non-adherent therapists feel responsible for poor outcome [32] or families feel responsible for being treatment failures [33]. An alternative hypothesis is perhaps that strict application of a treatment manual may lead to treatment failures. Rather than lying with clinicians or families, the problem may lie with how manuals are taught, implemented and supervised.

It has been repeatedly demonstrated that rates of the adoption of evidence-based manuals by clinicians in the eating disorders field are low. For those practitioners that identify as using manuals, adherence to the manual is also low [34–38]. One study of 40 therapists delivering FBT to young people with AN found not one therapist delivered a therapy that was consistently adherent to the FBT manual [34]. Similarly, another study found that around one third of FBT practitioners deviated substantially from manual adherent treatment [39]. Research has shown that CBT for adults with eating disorders is also commonly delivered in ways that deviate substantially from empirically-supported protocols, with fewer than half of self-defined CBT clinicians using core CBT techniques when delivering treatment [27]. These studies do not discuss if non-adherent practitioners have better or worse outcomes than adherent practitioners.

### Why do therapists deviate from manuals?

Potential reasons cited for non-adherence and therapist drift include comorbidities in complex cases, when the patient is perceived as treatment resistant, a lack of training and supervision in the protocol, and therapist preference, anxiety, mood or personality [26, 38]. These reasons are often framed as the therapist prioritising their own needs or preferences over those of their patient. However, a difficulty with maintaining strict adherence to manuals is that some research indicates that the inclusion of key aspects of treatment manuals does not necessarily impact outcome, such as including the family meal in session two [40, 41] or siblings attending FBT [42, 43]. One implication of this is that adaption of manuals, rather than strict adherence, need to be considered in our training and supervision of the use of manuals in clinical practice. Research into adaptations of manualised family therapy treatments has demonstrated promising results. For example, Parent-Focused Treatment (PFT) [42], short verses longer form of FBT [44], Multi-family therapy (MFT) [45, 46], Temperament-Based Therapy with Supports (TBT-S) [47], adapted family admissions [48], brief intensive programmes [49] and carer skills training programs [50]. Given full recovery rates at end of FBT (as measured by > 95% BMI and within one standard deviation of average Eating Disorder Examination (EDE) Global scores) are between 22 and 49% [42, 44, 51], with dropout rates of up to 20% [42, 52], the need to continue developing appropriate adaptations to existing manuals is something which the authors of manuals themselves, as well as the field more generally, acknowledge and promote [53, 54].

It is worth noting that most treatment manuals include statements promoting adaptations and acknowledging that this is required to apply the treatment most effectively in an individualised and real-world clinical context. This is important as clinical research needs to have real-world significance in addition to research based significance. While comorbid psychological disorders like anxiety and depression often mean a person may be excluded from a treatment trial, the reality of clinical work is that the majority of patients will present with comorbidities which may, and should, impact treatment [55–57]. Furthermore, adaptation of treatment manuals is often crucial to respectfully and sensitively embrace different cultural backgrounds of the patient, families, and therapist. More recent research has also identified the need to adapt treatment to better work for various populations, including young people or their families who identify as neurodiverse, such as those diagnosed with Autistic Spectrum Disorder [58], young people engaging in self-harm [59], or those who experience gender diversity or dysphoria [60]. Adaptation is also crucial in working in a trauma informed manner, where issues such as attachment and interpersonal style, self-concept and capacity for mentalisation are important considerations [61].

Interestingly, therapists with greater clinical experience are more likely to deviate from treatment manuals, which may be explained by the finding that some therapists view manuals as too inflexible to apply appropriately to complex cases [62, 63]. This may also be a result of applying evidence-informed practice, which promotes a flexible process of balancing best clinical evidence with therapist experience or clinical opinion and patient values and preferences [64, 65]. Therapists newer to a specific model cite a number of reasons why they are less adherent, including inadequate consideration of comorbid conditions, difficulties engaging parents or members of the multidisciplinary team and insufficient training and supervision [24, 66].

### Does adherence lead to better outcomes?

When manual adherence is low, it makes sense that outcomes differ from those found in clinical trials, which inherently require a high degree of manual adherence [28, 67]. A review of the wider psychotherapy literature by those in the eating disorders field allows consideration of whether strict adherence to any treatment manual is of benefit to our patients. In reality, it remains completely unsubstantiated whether or not high levels of adherence are related to therapeutic outcome [68, 69]. A meta-analysis of 36 treatment integrity outcome studies in adults concluded that neither manual adherence nor competence, defined as how well interventions were delivered, displayed an effect on outcome that was significantly different from zero [70]. A review of literature in child and adolescent psychotherapy found a small relationship between adherence and outcome in a meta-analysis of 35 studies [71]. However, the authors note that “the finding of a statistically significant association with outcome is tempered by the fact that the small effect suggests that adherence only accounts for just under one percent of variance in outcomes” (p.426). Importantly, neither therapeutic alliance nor patient readiness for change were considered as potential moderating variables. There is also some data that therapists being flexible in adherence may lead to improved treatment effects, at least within psychodynamic psychotherapy [72, 73].

There is an increasing amount of research on manual adherence relating to treatment efficacy within eating disorders specifically. Loeb et al. [74] found no significant association between adherence and outcome in either CBT or IPT for Bulimia Nervosa (BN). Another study found a complex relationship between adherence and outcome in CBT which changed over time [75]. Initial adherence was high in the behaviourally focused early and middle sessions, with therapists becoming less adherent in later sessions. While higher early adherence was related to less binging behaviour, this correlation decreased over time. There was also a notable correlation between high early adherence and increased drop out [75]. Interestingly, while it is noted that there was significant variation in adherence between the four therapists in the study, it is not reported if the less adherent therapists had poorer outcomes. One limitation of this study is the small sample size, although it does highlight an interesting area for future investigation. There is also a need to research the relationship between adherence and outcome in treatment for AN.

### Considering complexity: moderating factors

Perhaps not surprisingly, there appears to be a more complex relationship between adherence and outcome than typically assumed. Potential moderators of the relationship between adherence and outcome may include type of therapy (multisystemic or individual), patient diagnosis, therapist competence, therapeutic alliance and patient readiness to change [76].

Within the general psychotherapy literature, it appears that rigidly implementing a therapy approach is ineffective at best and potentially deleterious to outcomes at worst [77]. This is supported by research which suggests that where therapeutic alliance is poor, strong manual adherence is associated with a poorer outcome [76]. One study found that manual adherence to treat panic disorder was unhelpful when patients had low readiness for change, but was not related to outcome in patients with higher levels of motivation [78]. In evaluating CBT for adults with depression, Snippe et al. [79] found that therapist adherence was not related to positive outcome, particularly when patients were less engaged in treatment. Barber et al. [80] found that therapist adherence when working with people with substance abuse difficulties was less strongly related to outcome when therapeutic alliance was strong. Weck et al. [81] found that in three RCTs for depression, social phobia and hypochondriasis, alliance at session one, but not session two, predicted therapist adherence in later sessions, but that adherence had no relationship with future alliance. Such studies also demonstrate that therapeutic relationship and adherence are likely interrelated and bidirectional.

The above research highlights the complex interaction between patient readiness to change, manual adherence and outcome. When patients have low readiness to change, strict adherence to an action-based manual is likely to be ineffective [82]. This is especially pertinent to patients with AN, characterised by its ego syntonic nature in which sustained readiness to change is typically low [83]. It follows that an initial focus of treatment may therefore need to be engagement and building therapeutic alliance and increasing a patient’s readiness to change, rather than working strictly to a manual [84]. One of the major strengths behind family therapy for eating disorders is that even though young people may not want to engage in treatment, parents are often appropriately concerned and highly motivated. However, this by no means negates the importance of engaging the young person in the process. Strategies to build rapport and engage young people are being developed, such as using psychoeducation on the biological effects of starvation (for example, delayed gastric emptying) [11, 85].

While the relationship between adherence and outcome remains unclear, therapeutic alliance consistently predicts, or is at least correlated with, treatment outcome [86]. Baier et al. [87] reports that there are now over 300 studies of the alliance-outcome relationship, with stronger alliance consistently associated with positive treatment outcome across a range of psychotherapies. It is proposed that alliance is a non-specific mediator of change and is important in all psychotherapies [88], with the impact of the therapist relationship on patient outcome estimated to be between 5 and 8% [89]. Similarly, another study found that 97% of variance between the outcome of individual therapists was due to their ability to form better therapeutic relationships with patients [90]. Of course, it is also likely that alliance and adherence are interrelated and reinforce one another during therapy [89].

Parallel processes are a well-documented phenomenon in which therapists, supervisors, multidisciplinary teams and researchers can unknowingly begin to emulate and repeat the same patterns present for their patients [91, 92]. For an illness such as AN, which is characterised by cognitive and behavioural rigidity, higher threat sensitivity and intolerance of uncertainty, we must be careful as a field that we do not unknowingly fall into the same rigid processes. An example of this may be a specialist treatment team who refuses to provide treatment to a 15 year old patient with AN seeking help without support of their family because they strictly adhere to the idea that family therapy is the only appropriate option, despite the NICE guidelines offering suitable evidence-based alternatives.

### Implications for training and supervision

The positive impact of alliance on patient outcome, seemingly over manual adherence, is demonstrated both in the eating disorder and wider psychotherapy literature. Additionally, the importance of readiness to change on the appropriateness of manual adherence is also substantiated. As such, this paper questions the focus on strict manual adherence in training and supervision in the eating disorders field. While there remain numerous benefits to the development and application of treatment manuals as previously mentioned, the field may be better served by shifting the emphasis of training and supervision away from strict adherence to a more flexible, yet empirically consistent approach. Perhaps one can envisage manuals in the eating disorders field as a series of core interventions or tasks that should be included in an eating disorders treatment. Recent practice and training standards [7] have alluded to this way of thinking about treatment. These guidelines highlight that some of the core principles of eating disorder treatment may include developing a therapeutic relationship, assessing motivational status, the use of “evidence-based interventions” which include weighing the patient, a focus on modifying the eating disordered behaviour, developing a clinical case formulation and developing alternative coping strategies, amongst others. These guidelines are clearly based on the existing manuals and evidence base, but perhaps suggest a core principle approach rather than strict manual adherence.

One potential method to move away from a stirictly manualised approach to clinical training and practice has been described through Process Oriented Therapy (POT) [93]. POT proposes that treatment, training and supervision should focus on core clinical processes, including motivational enhancement, behavioural activation, cognitive flexibility and emotional regulation skills, rather than manualised content. In this view, training and supervision should focus on identifying specific patient needs through individual and systemic case formulation, rather than the application of specific manuals. One core process discussed is psychological flexibility, as opposed to rigidity. This seems especially important when working to increase psychological flexibility in people with AN. Further research, clinical work and training is already exploring the importance of promoting flexibility in over-controlled individuals through therapeutic adaptations like Radically Open Dialectical Behaviour Therapy (RO-DBT) [94]. Interestingly, POT also highlights the role of the therapeutic relationship and suggests this may model, instigate and promote the internalisation of an ability to be within the present moment, accept difficult experiences and engage in valued action [93].

Constantino et al. [76] offer a further suggestion to rethinking our training and supervision process. They describe Context Responsive Psychotherapy Integration (CRPI) as another alternative to strictly adherent manual training. The authors draw on data suggesting that successful management of alliance ruptures predicts treatment outcome [95], and therefore that training and supervision should help a therapist to identify and respond to the dynamic nature of the therapeutic relationship. They suggest that adjusting therapist actions to patients’ presenting difficulties, context and non-diagnostic characteristics, including the interpersonal dynamics present during therapy, represents a more skilful and nuanced approach that is well supported within evidence-informed practice. They also suggest replacing long-form trainings on single treatment manuals for specific diagnostic problems with briefer trainings that focus on the main principles underlying the manuals. Training would also dedicate time to teaching the clinicians important contextual markers, such as when to move away from the manual (for example, patient hesitancy to change or need to further develop clinical formulation) and empirically supported interventions appropriate for that circumstance (for example, use motivational enhancement techniques) [84].

Adoption of a more flexible core principle approach to treatment has implications for how adherence is assessed. We are in no way calling for an abandonment of treatment manuals or randomised controlled trials evaluating them. These trials, which need strict adherence to assist replicability, provide important data on which techniques should be included in a core principle approach (for example, weighing the patient). The document produced by Hurst et al. [7] could be modified into a checklist form, so that clinicians can assess their adherence to the core principles in treatment. It may also be important to assess common factors of therapy, such as the therapist’s ability to be empathic and to form a therapeutic alliance, as well as specific techniques included in the treatment. Of course, the key assessment is to monitor patient outcome on a regular basis [7]. Assessment of treatment acceptability, in either a strict or flexible form, to both therapist and patient may also be important.

## Conclusion

This paper recognises the important advances that treatment manuals have provided our field. Manuals promote rigorous research, provide clear training and clinical guidelines, allow broader dissemination and accessibility, and create a common language and platform from which to research necessary adaptations. Taken together, manuals provide a set of core principles important in the evidence-informed treatment of eating disorders, such as monitoring weight and other markers of health, supporting physical recovery as a means to establish psychological recovery, and utilising a patient’s support network. We know that training in the delivery of manualised treatment does make the clinician more adherent to the delivery of that manualised treatment [69]. However, a growing body of literature from both the field of general psychotherapy and within eating disorders specifically indicates that treatment adherence does not consistently predict patient outcomes and may indeed be contraindicated in some cases. We suggest that clinicians be encouraged in their clinical practice, training and supervision to use the treatment manual alongside individual and systemic case formulation, the client’s stage of change and the therapeutic relationship, with a focus on resolving therapeutic breaches. In combination, these factors allow a clinician to truly engage in evidence-informed practice. We would recommend future research is undertaken regarding formulation-driven adaptation of manuals, and training and supervision that goes beyond strict adherence to treatment manuals in the field of eating disorders.

## Data Availability

Not applicable.

## Abbreviations

AN: Anorexia Nervosa
FBT: Family-Based Treatment
CBT-E: Enhanced Cognitive Behaviour Therapy
NICE: National Institute for Health and Care Excellence
FT-AN: Family therapy for Anorexia Nervosa
AFT: Adolescent Focused Therapy
CBT: Cognitive Behaviour Therapy
MANTRA: Maudsley Anorexia Nervosa Treatment for Adults
SSCM: Specialist Supportive Clinical Management
FFP: Focal Psychodynamic Psychotherapy
IPT: Interpersonal Psychotherapy
RCT: Randomised Controlled Trial
DSM: Diagnostic and Statistical Manual of Mental Disorders
PFT: Parent-Focused Treatment
MFT: Multi-Family therapy or Multiple Family Therapy
TBT-S: Temperament-Based Therapy with Supports
EDE: Eating Disorder Examination
BN: Bulimia Nervosa
POT: Process Oriented Therapy
ACT: Acceptance and Commitment Therapy
RO-DBT: Radically Open Dialectical Behaviour Therapy
CRPI: Context Responsive Psychotherapy Integration

## References

[1] Treasure J, Duarte TA, Schmidt U (2020). Eating disorders. Lancet.

[2] Lock J, Le Grange D, Agras WS, Dare C (2000). Treatment manual for anorexia Nervosa: a family-based approach.

[3] Lock J, Le Grange D (2012). Treatment manual for anorexia nervosa: a family-based approach.

[4] Fairburn CG (2008). Cognitive behaviour therapy and eating disorders.

[5] Waller G, Cordery H, Corstorphine E, Hinrichsen H, Lawson R, Mountford V, Russell K (2007). Cognitive behavioural therapy for eating disorders: a comprehensive treatment guide.

[6] Heruc G, Hurst K, Casey A (2020). ANZAED eating disorder treatment principles and general clinical practice and training standards. J Eat Disord.

[7] Hurst K, Heruc G, Thornton C (2020). ANZAED practice and training standards for mental health professionals providing eating disorder treatment. J Eat Disord.

[8] Heruc G, Hart S, Stiles G (2020). ANZAED practice and training standards for dietitians providing eating disorder treatment. J Eat Disord.

[9] National Institute for Clinical Excellence. Eating disorders: recognition and treatment NG69 2017. Available online: https://www.nice.org.uk/guidance/ng69. Accessed 13 May 2020.

[10] Hilbert A, Hoek HW, Schmidt R (2017). Evidence-based clinical guidelines for eating disorders: international comparison. Curr Opin Psychiatry.

[11] Eisler I, Simic M, Blessitt E, Dodge L (2019). Maudsley service manual for child and adolescent eating disorders.

[12] Grave RD, Calugi S (2020). Cognitive behaviour therapy for adolescents with eating disorders.

[13] Lock D (2020). Adolescent-focused therapy for anorexia nervosa: a developmental approach.

[14] Schmidt U, Wade T, Treasure J (2014). The Maudsley Model of Anorexia Nervosa Treatment for Adults (MANTRA): development, key features, and preliminary evidence. J Cogn Psychother.

[15] McIntosh VV, Jordan J, Luty SE, Carter FA, McKenzie JM, Bulik CM, Joyce PR (2006). Specialist supportive clinical management for anorexia nervosa. Int J Eat Disord.

[16] Friederich H, Wild B, Zipfel S, Schauenburg H, Herzog W (2019). Anorexia Nervosa: focal psychodynamic psychotherapy.

[17] Zipfel S, Wild B, Gross G, Friederich H, Teufel M, Schellberg D (2014). Focal psychodynamic therapy, cognitive behaviour therapy, and optimised treatment as usual in outpatients with anorexia nervosa (ANTOP study): randomised controlled trial. Lancet.

[18] Carter FA, Jordan J, McIntosh VV, Luty SE, McKenzie JM, Frampton CM, Bulik CM, Joyce PR (2011). The long-term efficacy of three psychotherapies for anorexia nervosa: a randomized, controlled trial. Int J Eat Disord.

[19] Murray SB, Quintana DS, Loeb KL, Griffiths S, le Grange D (2019). Treatment outcomes for anorexia nervosa: a systematic review and meta-analysis of randomized controlled trials. Psychol Med.

[20] Lock J, Kraemer H, Jo B, Couturier J (2019). When meta-analyses get it wrong: Response to ‘treatment outcomes for anorexia nervosa: a systematic review and meta-analysis of randomized controlled trials’. Psychol Med.

[21] Greenhalgh T (2010). How to read a paper: the basics of evidence-based medicine.

[22] American Psychiatric Association. Diagnostic and statistical manual of mental disorders: DSM-5. Arlington: VA; American Psychiatric Association; 2013.

[23] Beumont P, Hay P, Beumont R (2003). Summary Australian and New Zealand clinical practice guideline for the management of Anorexia Nervosa. Australas Psychiatry.

[24] Waller G (2016). Treatment protocols for eating disorders: clinicians' attitudes, concerns, adherence and difficulties delivering evidence-based psychological interventions. Curr Psychiatry Rep.

[25] Cooper Z, Bailey-Straebler S (2015). Disseminating evidence-based psychological treatments for eating disorders. Curr Psychiatry Rep.

[26] Waller G, Mountford VA, Tatham M, Turner H, Gabriel C, Webber R (2013). Attitudes towards psychotherapy manuals among clinicians treating eating disorders. Behav Res Ther.

[27] Waller G, Stringer H, Meyer C (2012). What cognitive behavioral techniques do therapists report using when delivering cognitive behavioral therapy for the eating disorders?. J Consult Clin Psychol.

[28] Waller G, Turner H (2016). Therapist drift redux: why well-meaning clinicians fail to deliver evidence-based therapy, and how to get back on track. Behav Res Ther.

[29] Lavender KR (2020). Rebooting "failed" family-based treatment. Front Psych.

[30] Mulkens S, de Vos C, de Graaff A, Waller G (2018). To deliver or not to deliver cognitive behavioral therapy for eating disorders: Replication and extension of our understanding of why therapists fail to do what they should do. Behav Res Ther.

[31] Waller G (2009). Evidence-based treatment and therapist drift. Behav Res Ther.

[32] Aradas J, Sales D, Rhodes P, Conti J (2019). “As long as they eat”? Therapist experiences, dilemmas and identity negotiations of Maudlsey and family-based therapy for anorexia nervosa. J Eat Disord.

[33] Wufong E, Rhodes P, Conti J (2019). "We don't really know what else we can do": parent experiences when adolescent distress persists after the Maudsley and family-based therapies for anorexia nervosa. J Eat Disord.

[34] Couturier J, Kimber M, Jack S, Niccols A, Van Blyderveen S, McVey G (2013). Understanding the uptake of family-based treatment for adolescents with anorexia nervosa: therapist perspectives. Int J Eat Disord.

[35] Tobin DL, Banker JD, Weisberg L, Bowers W (2007). I know what you did last summer (and it was not CBT): a factor analytic model of international psychotherapeutic practice in the eating disorders. Int J Eat Disord.

[36] Wallace LM, von Ranson KM (2012). Perceptions and use of empirically-supported psychotherapies among eating disorder professionals. Behav Res Ther.

[37] Wallace LM, von Ranson KM (2011). Treatment manuals: Use in the treatment of bulimia nervosa. Behav Res Ther.

[38] Simmons AM, Milnes SM, Anderson DA (2008). Factors influencing the utilization of empirically supported treatments for eating disorders. Eat Disord.

[39] Kosmerly S, Waller G, Lafrance RA (2015). Clinician adherence to guidelines in the delivery of family-based therapy for eating disorders. Int J Eat Disord.

[40] Herscovici CR, Kovalskys I, Orellana L (2017). An exploratory evaluation of the family meal intervention for adolescent Anorexia Nervosa. Fam Process.

[41] Agras WS, Lock J, Brandt H, Bryson SW, Dodge E, Halmi KA, Jo B, Johnson C, Kaye W, Wilfley D, Woodside B (2014). Comparison of 2 family therapies for adolescent anorexia nervosa: a randomized parallel trial. JAMA Psychiat.

[42] le Grange D, Hughes EK, Court A, Yeo M, Crosby RD, Sawyer SM (2016). Randomized clinical trial of parent-focused treatment and family-based treatment for adolescent anorexia nervosa. J Am Acad Child Adolesc Psychiatry.

[43] Ellison R, Rhodes P, Madden S, Miskovic J, Wallis A, Baillie A, Kohn M, Touyz S (2012). Do the components of manualized family-based treatment for anorexia nervosa predict weight gain?. Int J Eat Disord.

[44] Lock J, Agras WS, Bryson S, Kraemer HC (2005). A comparison of short- and long-term family therapy for adolescent anorexia nervosa. J Am Acad Child Adolesc Psychiatry.

[45] Eisler I (2005). The empirical and theoretical base of family therapy and multiple family day therapy for adolescent anorexia nervosa. J Fam Ther.

[46] Eisler I, Simic M, Hodsoll J, Asen E, Berelowitz M, Connan F, Ellis G, Hugo P, Schmidt U, Treasure J, Yi I, Landau SA (2016). Pragmatic randomised multi-centre trial of multifamily and single family therapy for adolescent anorexia nervosa. BMC Psychiatry.

[47] Kaye W, Wierenga C, Knatz S, Liang J, Boutelle K, Hill L, Eisler I (2014). Temperament-based treatment for anorexia Nervosa. Eur Eat Disord Rev.

[48] Wallis A, Alford C, Hanson A, Titterton J, Madden S, Kohn M (2013). Innovations in Maudsley family-based treatment for anorexia nervosa at the Children's Hospital at Westmead: a family admission programme. J Fam Ther.

[49] Knatz S, Murray SB, Matheson B, Boutelle KN, Rockwell R, Eisler I, Kaye WH (2015). A brief, intensive application of multi-family-based treatment for eating disorders. Eat Disord.

[50] Hodsoll J, Rhind C, Micali N (2017). A Pilot, Multicentre pragmatic randomised trial to explore the impact of carer skills training on carer and patient behaviours: testing the cognitive interpersonal model in adolescent anorexia nervosa. Eur Eat Disord Rev.

[51] Madden S, Miskovic-Wheatley J, Wallis A, Kohn M, Lock J, Le Grange D, Jo B, Clarke S, Rhodes P, Hay P, Touyz S (2015). A randomized controlled trial of in-patient treatment for anorexia nervosa in medically unstable adolescents. Psychol Med.

[52] Dejong H, Broadbent H, Schmidt U (2012). A systematic review of dropout from treatment in outpatients with anorexia nervosa. Int J eat Disord.

[53] Lock J, Le Grange D, Agras WS, Moye A, Bryson SW, Jo B (2010). Randomized clinical trial comparing family-based treatment with adolescent-focused individual therapy for adolescents with anorexia nervosa. Arch Gen Psychiatry.

[54] Lock J, Le Grange D. Family-based treatment: Where are we and where should we be going to improve recovery in child and adolescent eating disorders. Int. J Eating Disord. 2018; 10.1002/eat.22980. 10.1002/eat.22980 30520532

[55] Rienecke RD (2017). Family-based treatment of eating disorders in adolescents: current insights. Adolesc Health Med Ther.

[56] Strober M, Johnson C (2012). The need for complex ideas in Anorexia Nervosa: why biology, environment, and psyche all matter, why therapists make mistakes, and why clinical benchmarks are needed for managing weight correction. Int J Eat Disorders.

[57] Hughes EK, Goldschmidt AB, Labuschagne Z, Loeb KL, Sawyer SM, le Grange D (2013). Eating disorders with and without comorbid depression and anxiety: similarities and differences in a clinical sample of children and adolescents. Eur Eat Disord Rev.

[58] Kinnaird E, Norton C, Stewart C, Tchanturia K (2019). Same behaviours, different reasons: what do patients with co-occurring anorexia and autism want from treatment?. Int Rev Psychiatry.

[59] Reilly EE, Orloff NC, Luo T, Berner LA, Brown TA, Claudat K, Kaye WH, Anderson LK (2020). Dialectical behavioral therapy for the treatment of adolescent eating disorders: a review of existing work and proposed future directions. Eat Disord.

[60] Diemer EW, Hughto JMW, Gordon AR, Guss C, Austin SB, Reisner SL (2018). Beyond the binary: differences in eating disorder prevalence by gender identity in a transgender sample. Transgender Health.

[61] Tasca GA (2019). Attachment and eating disorders: a research update. Curr Opin Psychol.

[62] Haas HL, Clopton JR (2003). Comparing clinical and research treatments for eating disorders. Int J Eat Disord.

[63] Mountford V, Tatham M, Turner H, Waller G (2017). Complexity in eating disorders: a case for simple or complex formulation and treatment?. Cognit Behav Ther.

[64] Sackett DL, Rosenberg WMC, Muir Gray JA, Haynes RB, Richardson W (1996). Evidence based medicine: what it is and what it isn't. BMJ.

[65] Nevo I, Slonim-Nevo V (2011). The myth of evidence-based practice: towards evidence-informed practice. Br J Soc Work.

[66] Couturier J, Kimber M, Jack S, Niccols A, Van Blyderveen S, McVey G (2014). Using a knowledge transfer framework to identify factors facilitating implementation of family-based treatment. Int J Eat Disord.

[67] Bearman SK, Schneiderman RL, Zoloth E (2017). Building an evidence base for effective supervision practices: an analogue experiment of supervision to increase EBT fidelity. Adm Policy Ment Health.

[68] Perepletchikova F, Kazdin AE (2005). Treatment integrity and therapeutic change: Issues and research recommendations. Clin Psychol Sci Pract.

[69] Miller SJ, Binder JL. The effects of manual-based training on treatment fidelity and outcome: a review of the literature on adult individual psychotherapy. Psychother Theory Res Pract Train. 2002; 10.1037/0033-3204.39.2.184

[70] Webb CA, Derubeis RJ, Barber JP (2010). Therapist adherence/competence and treatment outcome: a meta-analytic review. J Consult Clin Psychol.

[71] Collyer H, Eisler I, Woolgar M (2020). Systematic literature review and meta-analysis of the relationship between adherence, competence and outcome in psychotherapy for children and adolescents. Eur Child Adolesc Psychiatry.

[72] Katz M, Hilsenroth MJ, Gold JR, Moore M, Pitman SR, Levy SR, Owen J (2019). Adherence, flexibility, and outcome in psychodynamic treatment of depression. J Couns Psychol.

[73] Owen J, Hilsenroth MJ (2014). Treatment adherence: the importance of therapist flexibility in relation to therapy outcomes. J Couns Psychol.

[74] Loeb KL, Wilson GT, Labouvie E, Pratt EM, Hayaki J, Walsh BT, Agras WS, Fairburn CG (2005). Therapeutic alliance and treatment adherence in two interventions for bulimia nervosa: a study of process and outcome. J Consult Clin Psychol.

[75] Folke S, Daniel SIF, Gondan M, Lunn S, Tækker L, Poulsen S (2017). Therapist adherence is associated with outcome in cognitive-behavioral therapy for bulimia nervosa. Psychotherapy (Chic).

[76] Constantino MJ, Coyne AE, Muir HJ (2020). Evidence-based therapist responsivity to disruptive clinical process. Cognit Behav Pract.

[77] Castonguay LG (2011). Psychotherapy, psychopathology, research and practice: pathways of connections and integration. Psychother Res.

[78] Huppert J, Barlow D, Gorman J, Shear K, Woods S (2006). The interaction of motivation and therapist adherence predicts outcome in cognitive behavioral therapy for panic disorder: preliminary findings. Cognit Behav Pract.

[79] Snippe E, Schroevers MJ, Tovote KA, Sanderman R, Emmelkamp PMG, Fleer J (2019). Explaining variability in therapist adherence and patient depressive symptom improvement: the role of therapist interpersonal skills and patient engagement. Clin Psychol Psychother.

[80] Barber J, Gallop R, Crits-Christoph P, Frank A, Thase M, Weiss R, Gibbons M (2006). The role of therapist adherence, therapist competence, and alliance in predicting outcome of individual drug counseling: Results from the National Institute Drug Abuse Collaborative Cocaine Treatment Study. Psychother Res.

[81] Weck F, Grikscheit F, Jakob M, Höfling V, Stangier U (2015). Treatment failure in cognitive-behavioural therapy: therapeutic alliance as a precondition for an adherent and competent implementation of techniques. Br J Clin Psychol.

[82] Vitousek K, Watson S, Wilson GT (1998). Enhancing motivation for change in treatment-resistant eating disorders. Clin Psychol Rev.

[83] Bewell CV, Carter JC (2008). Readiness to change mediates the impact of eating disorder symptomatology on treatment outcome in anorexia nervosa. Int J Eat Disord.

[84] Macdonald P, Hibbs R, Corfield F, Treasure J (2012). The use of motivational interviewing in eating disorders: a systematic review. Psychiatry Res.

[85] Jewell T, Blessitt E, Stewart CS, Simic M, Eisler I (2016). Family therapy for child and adolescent eating disorders: a critical review. Fam Process.

[86] Horvath AO, Del Re AC, Flückiger C, Symonds D (2011). Alliance in individual psychotherapy. Psychotherapy.

[87] Baier AL, Kline AC, Feeny NC (2020). Therapeutic alliance as a mediator of change: a systematic review and evaluation of research. Clin Psychol Rev.

[88] Bertolino B, Bargmann S, Miller SD (2012). Manual 1: what works in therapy: a primer.

[89] Baldwin SA, Wampold BE, Imel ZE (2007). Untangling the alliance-outcome correlation: exploring the relative importance of therapist and patient variability in the alliance. J Consult Clin Psychol.

[90] Lange AM, van der Rijken RE, Delsing MJ, Busschbach JJ, van Horn JE, Scholte RH (2017). Alliance and adherence in a systemic therapy. Child Adolesc Ment Health.

[91] Mothersole G (1999). Parallel process. Clin Superv.

[92] Hamburg P, Herzog D (1990). Supervising the therapy of patients with eating disorders. Am J Psychother.

[93] Hayes SC, Hofmann SG, Ciarrochi J (2020). A process-based approach to psychological diagnosis and treatment: the conceptual and treatment utility of an extended evolutionary model. Clin Psychol Rev.

[94] Lynch TR, Hempel RJ, Dunkley C (2015). Radically open-dialectical behavior therapy for disorders of over-control: signaling matters. Am J Psychother.

[95] Eubanks CF, Sergi J, Samstag LW, Muran JC (2021). Commentary: Rupture repair as a transtheoretical corrective experience. J Clin Psychol.

